# Associations between online child sexual solicitation and abuse and offline child maltreatment: A latent class analysis

**DOI:** 10.1007/s00787-025-02885-5

**Published:** 2025-10-23

**Authors:** Katrin Chauviré-Geib, Ann-Christin Haag, Jelena Gerke, Cedric Sachser, Miriam Rassenhofer, Jörg M. Fegert

**Affiliations:** 1 Department of Child and Adolescent Psychiatry, Psychosomatics and Psychotherapy, University Hospital Ulm, Ulm, Germany; 2 German Centre for Mental Health (DZPG), partner site Mannheim-Heidelberg-Ulm, Germany; 3 Department of Psychology, Clinical Child and Adolescent Psychology, University of Bamberg, Bamberg, Germany; 4 Centre for Child Protection in Medicine, Baden-Württemberg, Germany; 5 Department of Child and Adolescent Psychiatry, Psychosomtics and Psychotherapy, University Hospital Ulm, Steinhövelstr. 5, 89075 Ulm, Germany

**Keywords:** Online child sexual solicitation and abuse (OCSSA), Child maltreatment, Child abuse and neglect (CAN), Adverse childhood experiences (ACE), SDG indicator 16.2.3

## Abstract

The increasing integration of digital technologies into daily life has led to a rise in online child sexual solicitation and abuse (OCSSA), posing significant risks to young people. Understanding how OCSSA is associated with other forms of offline child maltreatment is essential for improving diagnosis, treatment, and support in child and adolescent psychiatry. This study aims to identify the co-occurrence of OCSSA with other forms of offline child maltreatment experienced before the age of 18 in Germany. To most accurately assess the current reality among children and adolescents, the study surveyed a nationally representative sample of 1,006 participants in Germany, following indicator 16.2.3 of the Sustainable Development Goals, and assessed experiences of OCSSA and other forms of offline child maltreatment. Participants completed the Adverse Childhood Experiences Questionnaire and screening questions measuring OCSSA. Latent class analysis (LCA) was used to identify exposure profiles. The resulting subgroups were compared using Chi-Square-Tests and Mann-Whitney-U-Tests. The LCA revealed two classes: 85.39% of participants were classified as ‘predominantly OCSSA’ victims, while 14.61% experienced ‘poly-victimization’, primarily associated with OCSSA and emotional maltreatment. The two classes significantly differed regarding relationship status, education, and equivalence income. The findings highlight increased vulnerability to online victimisation in the digital age and emphasize the interplay between online and offline maltreatment, particularly for those who have experienced multiple forms of victimisation. They emphasize the need for early identification and improved risk assessment, early preventive strategies, and specific therapeutic interventions in child and adolescent psychiatric care.

Advancements in technology have established new environments and frameworks for certain forms of child sexual abuse (CSA), such as image-based sexual abuse, online grooming, sexual solicitations, sexual extortion (sextortion), or commercial extortions. These offences involve using technology to initiate, escalate, and maintain CSA. Online child sexual solicitation and abuse (OCSSA) is an overarching term that encompasses all forms of CSA in a digital context where the perpetrator is exclusively online and not physically present [1]. In such cases, the interaction remains entirely in digital spaces and there is no direct physical contact between the perpetrator and the victim. OCSSA represents a form of sexual abuse that falls under the category of ‘hands-off’ offences. It exists alongside other types of maltreatment, including physical abuse, emotional abuse, emotional neglect, and physical neglect. Growing up in an increasingly digital world, children and adolescents can be exposed to these risks in both online and offline environments. This dual exposure requires greater focus from both research and clinical practice, as professionals are likely to encounter affected individuals more frequently. One of the main objectives of the United Nations Sustainable Development Goals (SDGs) is to end violence against children, a goal that is specifically addressed in goal 16.2 [2]. It sets a clear target for ending all forms of violence against children and highlights the importance of monitoring trends in CSA. To facilitate effective monitoring, indicator 16.2.3 defines the 18- to 29-year-old age group as a key population for assessing experiences of violence occurring before the age of 18. This indicator group allows for a comprehensive global assessment of the realities faced by children and adolescents today.

Prevalence studies of OCSSA have increased in recent years. A prevalence of approximately 16.6% has been reported for children experiencing at least one form of OCSSA [3)]. A U.S. study was the first to examine various forms of OCSSA in a national sample. It was found, that 15.6% of participants experienced OCSSA, with unwanted sexual questions (18.3%) and unwanted sexual talk (16.9%) being reported predominantly [4]. A German representative study, using the same screening questions as the U.S. study and based on the same dataset as the present paper, found that 31.6% of young adults reported experiencing at least one form of OCSSA before the age of 18. The most commonly reported experiences included exposure to pornographic material (21.1%) and unwanted sexualized conversations (15.0%) [1]. In contrast, other forms of offline child maltreatment have been studied more extensively for years. International prevalence rates are particularly high for emotional abuse (36.3%) and physical abuse (22.6%) [5]. At the same time, research on child maltreatment is dominated by studies on sexual abuse [6]. Prevalence rates range from 6% to 18% [7]. However, these figures vary depending on the survey method, the gender of the victim, as well as living conditions and cultural perceptions, which may influence disclosure patterns.

The co-occurrence of different forms of child maltreatment is the rule rather than the exception, whether they occur simultaneously or sequentially [8–12]. Depending on sample size, survey methods, and instruments used, studies showed that up to 55% of individuals affected by child maltreatment experienced more than one type of maltreatment [13–15]. A meta-analysis by Matsumoto et al. [10] identified the most common overlaps between forms of child maltreatment: the most common overlap was between maltreatment in the form of threats, specifically physical and emotional abuse. This was followed by deprivation, which included both physical and emotional neglect, and emotional maltreatment, including emotional abuse and neglect. Individuals who experience multiple forms of child maltreatment are at increased risk of negative outcomes, as the presence of multiple types of adversity accumulates. Early adverse childhood experiences (ACEs), including forms of child maltreatment, have a profound impact on neurobehavioral personality development. Being exposed early to ACEs can affect health and well-being across the lifespan, potentially leading to neurodevelopmental disorders, social, emotional, and cognitive impairments, risky health behaviours, disease, disability, and social problems as well as premature death [16, 17]. This cumulative effect is often referred to in trauma research as the ‘building block effect’, where the likelihood of developing secondary diseases increases with the presence of different types of traumatic stressors, such as multiple stressful childhood experiences. This phenomenon is further illustrated by the dose-response relationship found in studies of child maltreatment [13], which indicates that children who experience multiple types of maltreatment have more severe negative mental health outcomes.

Understanding the co-occurrences of different forms of child maltreatment is critical, especially given the increasing prevalence of Internet use and the unique risk factors associated with the online environment. However, while previous research has examined patterns of co-occurrence among physical abuse, emotional abuse, sexual abuse, emotional neglect, and physical neglect [18], a systematic analysis of the co-occurrence of OCSSA with other forms of offline child maltreatment has not yet been conducted. It remains to be seen whether OCSSA has distinct patterns of co-occurrence with other forms of maltreatment or contributes to specific risk profiles for poly-victimization. Without a clearer understanding of these relationships, it is difficult to develop targeted strategies that address the full spectrum of victimization experiences, improving diagnosis, treatment, and support in child and adolescent psychiatry. Therefore, this study aims to systematically examine the co-occurrence of OCSSA with other forms of offline child maltreatment, with the goal of identifying patterns and therefore potential risk profiles associated with poly-victimization. An exploratory approach was used to investigate whether specific groups or risk profiles could be identified, without any prior hypotheses being formulated. Additionally, this study provides an overview of the prevalence rates of OCSSA and other forms of offline child maltreatment experienced during childhood and adolescence in the German population. Effective monitoring of emerging trends in childhood and adolescence requires the collection of data from individuals aged 18–29, as specified in indicator 16.2.3 of the SDGs, to allow insight to be gained across the entire period of childhood and adolescence [2]. As the SDGs call for a comprehensive global assessment of young people’s realities [19], the integration of this indicator in child and adolescent psychiatry research is critical to gaining deeper insight into the challenges and needs of children and adolescents.

## Method

### Sample

The sample consisted of *N* = 1,006 participants between the ages of 18 and 29 of which 50.8% were male. The average age of the sample was 23.65 years (*SD* = 3.51). The sample was representative of the German population between 18 and 29 regarding age, gender, and geographic region. Sociodemographic and -economic characteristics are presented in Table 1.

**Table 1:** Sociodemographic characteristics of the sample

	Total sample (*N* = 1,006) *n* (%)
Age (M, SD)	23.65 (3.51)
Gender	
Female	493 (49.0)
Male	511 (50.8)
Diverse	2 (0.2)
In a relationship	
Yes	393 (39.1)
No	595 (59.3)
Highest level of education	
Basic education	640 (63.7)
Advanced education	363 (36.2)
Equivalence income	
Below poverty risk threshold (< €1500)	209 (20.8)
Above poverty risk threshold (> €1500)	764 (76.1)

Note. Highest level of education: basic education = no school degree (yet), lower secondary school certificate, and polytechnical high school; advanced education = graduation from technical college, entrance qualification to college/university, and university degree

### Procedure

Data collection took place between October 2023 and April 2024. A representative sample of the German-speaking resident population aged 16 and over was surveyed by USUMA, a demographic consultancy company (www.usuma.com). The sampling procedure included sample regions from 258 regions in Germany, a random route procedure, supplemented by a quota sampling method (for selecting the households) and a Kish-Selection-Grid (for selecting the respective target person of the household): A starting address, referred to as the sample point, and a step interval for randomly selecting households were defined for each regional area. Following these instructions, data collectors recorded doorbell names based on the specified step interval and adhered to random route walking guidelines to achieve comprehensive sample coverage. Subsequently, the identified households were approached and invited to take part in the study. Following the main survey, an oversample was conducted for participants between 18 and 29 years old using the quota method. To ensure that results could be analysed for all participants, particularly by age group, cases in the oversample were adjusted to reflect the proportional representation of the age group in the main sample. As a result, the analysis of 18–29-year-olds includes all completed surveys, resulting in lower margins of error and more precise confidence intervals.

Out of a total of 6,371 households contacted, *N* = 3,126 participated in the survey. Reasons for non-participation were households not reached after four visits (9.0%), households refused to provide information (39.4%), target person not reached after four visits (1.0%), target person out of town (0.4%), target person ill and unable to attend interview (0.5%) and target person refused interview (9.1%). The present sample was random due to the data collection procedure. Despite non-response bias, the dataset remains representative. Additionally, the final sample is weighted. Micro census data on the German population were used to create the weighting factor. Data were collected in two parts. First, socio-demographic data were collected in face-to-face interviews. The associated self-completion questionnaire was then given to the participants in paper form. This was completed independently and then sealed in an envelope by the participant. The interviewer was on hand to help with any difficulties. This study was performed in line with the principles of the Declaration of Helsinki. Approval was granted by the Ethics Committee of the University of Leipzig (reference number 278/23-ek).

The present study only analysed data from participants between the ages of 18 and 29 years, as this age group is particularly relevant to indicator 16.2.3 of the SDG, which aims to ensure that all young people are free from violence and exploitation [2]. By focusing on this population, this study seeks to monitor recent trends and developments in their experiences.

## Measures

Sociodemographic data were collected, including gender, age, relationship status, and education level. An estimation of the equivalence income was calculated, by dividing the net household income by the square root of the household size [20]. Education was dichotomized as basic education, including no school degree (yet), lower secondary school certificate, and polytechnical high school, and advanced education, including graduation from technical college, entrance qualification to college/university, and university degree. Equivalence income was dichotomized into below poverty risk threshold and above poverty risk threshold. The threshold was set at 60% of the median equivalized income.

The prevalence of maltreatment was assessed using the German version of the *Adverse Childhood Experiences Questionnaire* [21]. It is a reliable and valid screening instrument to measure the occurrence of various adverse childhood experiences [22,23], including five items each on child maltreatment and family dysfunction. The items have a dichotomous response format (yes/no) and aim to record the number of different stressful experiences. Thus, a higher total score indicates a higher number of adverse childhood experiences. The German version of the ACE Questionnaire demonstrated satisfactory psychometric properties, with an internal consistency of Cronbach’s α = 0.76 [23]. In our sample, Cronbach’s alpha was α = 0.77, showing a comparable level of reliability. For the present study, only the five items on child maltreatment, namely physical abuse, emotional abuse, hands-on sexual abuse, physical neglect, and emotional neglect were included, while household dysfunction items were not. Although household dysfunction is an important aspect, it represents indirect influences on a child’s environment rather than directly capturing the experience of abuse and neglect. In our sample, Cronbach’s alpha for the five child maltreatment items included was α = 0.70, reflecting acceptable internal consistency for this subset.

To measure *online child sexual solicitation and abuse* (OCSSA), this study employed screening questions originally developed by Finkelhor et al. [4]. These were translated into German and extended by an additional item on exposure to pornographic material which is relevant to the German legal context (see Table 2). The screening questions encompass 11 items on OCSSA. They have a dichotomous response format (no/yes) and aim to record the number of different experiences. Thus, a higher total score indicates a higher number of OCSSA experiences. The screening questions demonstrated acceptable psychometric properties, with an internal consistency of Cronbach’s α = 0.72. This study provides the first representative assessment of OCSSA in Germany.

**Table 2:** Questions assessing different forms of online child sexual abuse adapted from Finkelhor et al. [4]

OCSSA1	Has someone ever shared with other people a sexual picture or video of you without your permission before the age of 18?
OCSSA2	Has someone ever taken or made a sexual picture or video of you without your permission before the age of 18?
OCSSA3	Has someone ever threatened, tried to force you, or strongly pressured you to provide sexual pictures or videos online or through a cell phone before the age of 18?
OCSSA4	Has someone ever threatened to share a sexual picture or video of you to get you to do something— like take or send other sexual pictures of yourself, have a sexual relationship with them, pay them money, or something else before the age of 18?
OCSSA5	Did anyone ever use the internet or a cell phone to try to get you to talk about sex when you did not want to before the age of 18?
OCSSA6	Did anyone ever use the internet or a cell phone to ask you for sexual information about yourself when you did not want to answer those questions? This means very personal questions, like what your body looks like or sexual things you have done before the age of 18?
OCSSA7	Did anyone ever use the internet or a cell phone to ask you to do something sexual that you did not want to do before the age of 18?
	Have you done any of the following things over the internet or a cell phone (including texting) in exchange for money, drugs, or other valuable items before the age of 18?
OCSSA8	sexual talk
OCSSA9	making, sending, or posting sexual pictures or videos of oneself
OCSSA10	any other sexual activity
OCSSA11	Have you ever had unwanted contact with sexual or pornographic material on the Internet or via mobile phone before the age of 18?

### Statistical analysis

Descriptive statistics regarding the experiences of OCSSA and child maltreatment, as well as gender differences, were calculated using (exact) Chi-Square-Tests (*χ*^*2*^) and Mann-Whitney-U-Tests. Effect sizes were measured by Cramer’s V, with *V* = 0.1 representing small effects, *V* = 0.3 medium effects, and *V* = 0.5 large effects [24]. These analyses were conducted using SPSS (Version 29.0).

Latent class analysis (LCA) was conducted to identify profiles of poly-victimization, using R software (Version 4.4.1) and the *poLCA* [25], *lcmm* [26], and *e1071* [27] packages. LCA was chosen because it enables the identification of unobserved subgroups within a population based on concurrent experiences. It was used to identify latent subgroups based on the co-occurrences between offline child maltreatment and OCSSA. The aim of LCA was to determine how many subgroups, i.e., latent classes, there are, and to which class the participants belong [28, 29]. The best fitting model was identified starting at one class and testing up to six classes. The selection of the best fitting model was based on multiple criteria: (1) Statistical fit indices including corrected Akaike Information Criteria (cAIC), Bayesian Information Criteria (BIC), and adjusted Bayesian Information Criteria (aBIC), with lower values indicating a better fit [30]. The cAIC uses a sample size correction to balance model fit and complexity more effectively, thereby reducing overfitting. It also serves as a practical criterion for selecting parsimonious models [31]. (2) Entropy was considered acceptable above 0.8, with values closer to 1.0 indicating clearer class separation and better assignment of individuals to latent classes [32]. (3) The Lo-Mendell-Rubin likelihood ratio test (LMR-LRT) and Bootstrapped likelihood ratio test (BLRT) were used for comparing nested models, where a significant *p*-value indicates that the *k*-class model provides a better fit than the *k-1*-class model [33]. Nylund et al. [34] state that the BLRT performs better than other likelihood-ratio and information-theoretic statistical testing methods when the outcomes of these two likelihood ratio tests diverge. To avoid potentially unstable solutions, classes with a prevalence of less than 5% were excluded, as simulation studies [35] have shown that such rare classes are difficult to replicate [28]. (4) The plausibility and interpretability of classes were also considered. Next, sociodemographic information was compared across class membership using (exact) Chi-Square-Tests (*χ2*) and Mann-Whitney-U-Tests to describe only the characteristics of each class. Because the percentage of missing data for all variables included in the present study was very low (0.3%), missing data were not imputed for analyses.

## Results

### Descriptive statistics

In this study, 32.3% (*n* = 325) of participants had experienced at least one form of OCSSA before their 18th birthday. Of these, 52.6% (*n* = 171) were female, 47.1% (*n* = 153) were male and 0.3% (*n* = 1) identified as diverse. On average, participants reported having experienced 2.27 (*SD* = 1.45, min = 1, max = 8) forms of OCSSA. The median was 2. By gender, males had experienced 1.77 (*SD* = 1.09, min = 1, max = 6) forms of OCSSA with a median of 1. Females had experienced 2.72 (*SD* = 1.58, min = 1, max = 8) forms of OCSSA, the median was 3. The difference regarding the number of types of OCSSA experienced between male and female participants was significant with a small effect size (*U* = 136798.000, *Z* = 2.848, *p* =.001, *r* =.089). Experiences of unwanted confrontation with pornographic or sexualized material (22.1%), unwanted sexualized talk (16.0%), and unwanted sexualized questions (13.5%) were reported most frequently by the sample. Significant gender differences were found for non-consensual sharing of sexualized material, forced sexualized material, sextortion, unwanted sexualized talk, unwanted sexualized questions, and sexual requests for unwanted sexual acts with female participants being affected more frequently than male participants (Table 3). However, the effect sizes were small. There was no significant difference between male and female participants in experiencing at least one form of OCSSA. For more details, see Table 3.

**Table 3:** Forms of online child sexual solicitation and abuse across victim gender

Online child sexual solicitation and abuse	Total (*N* = 1006)	Male (*n* = 511)	Female (*n* = 493)	Chi^2^-Test
	*n* (%)	*n* (%)	*n* (%)	
Any form of OCSSA	324 (32.3)	153 (29.9)	171 (34.7)	*χ*^*2*^(1) = 2.58, *p* =.108 *V* = 0.051
**Non-consensual sharing of sexualized material**	**26 (2.6)**	**7 (1.4)**	**19 (3.9)**	***χ***^***2***^**(1) = 6.09**, ***p*** **=.014** ***V*** **= 0.078**
Non-consensual taking/making of sexualized material	33 (3.3)	12 (2.4)	21 (4.3)	*χ*^*2*^(1) = 2.91, *p* =.088 *V* = 0.054
**Forced sexualized material**	**41 (4.1)**	**11 (2.2)**	**30 (6.1)**	***χ***^***2***^**(1) = 9.71**, ***p*** **=.002** ***V*** **= 0.099**
**Sextortion**	**33 (3.3)**	**10 (2.0)**	**23 (4.7)**	***χ***^***2***^**(1) = 5.76**, ***p*** **=.016** ***V*** **= 0.076**
**Unwanted sexualized talk**	**160 (16.0)**	**53 (10.4)**	**107 (21.7)**	***χ***^***2***^**(1) = 23.94**, ***p*** **<.001** ***V*** **= 0.155**
**Unwanted sexualized questions**	**135 (13.5)**	**40 (7.9)**	**95 (19.3)**	***χ***^***2***^**(1) = 27.97** ***p*** **<.001** ***V*** **= 0.167**
**Request to engage in unwanted sexual acts**	**78 (7.8)**	**29 (5.7)**	**49 (10.0)**	***χ***^***2***^**(1) = 6.29**, ***p*** **=.012** ***V*** **= 0.079**
Commercial sexual talk	5 (0.5)	1 (0.2)	4 (0.8)	*χ*^*2*^(1) = 1.90, *p* =.168 *V* = 0.044
Commercial sexualized material	1 (0.1)	0	1 (0.2)	*χ*^*2*^(1) = 1.03, *p* =.492 *V* = 0.032
Commercial other sexual activity	3 (0.3)	0	3 (0.6)	*χ*^*2*^(1) = 3.11, *p* =.119 *V* = 0.056
Unwanted confrontation with pornographic/sexualized material	221 (22.1)	108 (21.2)	113 (22.9)	*χ*^*2*^(1) = 0.42, *p* =.516 *V* = 0.021

Note. OCSSA = online child sexual solicitation and abuse; *χ2* = Chi^2^; *V* = Cramer’s V; Bold indicates statistical significance at *p* <.050

Among the participants, 18.9% (*n* = 189) had experienced at least one form of child maltreatment. Of these, 51.3% (*n* = 97) were female and 48.7% (*n* = 92) were male. On average, victims had experienced 1.89 (*SD* = 0.98, min = 1, max = 5) types of child maltreatment. The median was 2. By gender, males had experienced 1.03 (*SD* = 0.92, min = 1, max = 4) types of child maltreatment, with a median of 2. Female victims had experienced 1.87 (*SD* = 1.03, min = 1, max = 5) types of child maltreatment, the median was 2. The difference regarding the number of types of child maltreatment experienced between male and female victims was not significant (*U* = 126758,000, *Z* = 0.577, *p* =.564). Females had experienced hands-on sexual abuse significantly more frequently than males, whereas there were no gender differences in either emotional and physical abuse or neglect. Table 4 depicts the comparison of child maltreatment events across gender.

**Table 4:** Forms of child maltreatment across gender

Child maltreatment	Total (*n* = 1006)	Male (*n* = 511)	Female (*n* = 493)	Chi^2^-Test
	*n* (%)	*n* (%)	*n* (%)	
Any form of child maltreatment	189 (18.9)	92 (18.2)	92 (19.8)	*χ2* = 0.461, *p* =.497 *V* = 0.021
Emotional abuse	132 (13.1)	69 (13.5)	63 (12.8)	*χ2* = 0.418, *p* =.811 *V* = 0.020
Physical abuse	67 (6.7)	41 (8.0)	26 (5.3)	*χ2* = 3.195, *p* =.202 *V* = 0.056
**Hands-on sexual abuse**	**35 (2.5)**	**9 (1.8)**	**26 (5.3)**	***χ2*** **= 9.329**, ***p*** **=.009** ***V*** **= 0.096**
Emotional neglect	107 (10.6)	52 (10.2)	55 (11.2)	*χ2* = 0.492, *p* =.782 *V* = 0.022
Physical neglect	20 (2.0)	9 (1.8)	11 (2.2)	*χ2* = 0.320, *p* =.852 *V* = 0.018

Note. *χ2* = Chi2; *V* = Cramer’s V; Bold indicates statistical significance at *p* <.050

### Identification of OCSSA co-occurrences

A two-class solution proved to be the best-fitting model. Table 5 shows the model fit for the class solutions. The BLRT was significant only for a two-class solution (*p* =.006), whereas the LMR-LRT was significant for a two-class, a three-class, and a four-class solution (*ps* < 0.001). As the BLRT outperforms the LMR-LRT as a more reliable indicator of class structure, the final decision was made based on its results. Both cAIC and BIC are the lowest for a two-class solution, and the entropy has the highest value. Although the aBIC is lowest for a four-class solution, a two-class model is preferred for its overall fit. The size of the smallest class in a two-class solution is also considered acceptable, reinforcing its robustness. In addition, the interpretation of the two classes makes this solution more meaningful and easier to apply in practice. In total, the statistical indicators and content consistency suggest that a two-class solution provides the clearest and most stable separation of the data, thereby enhancing the reliability of the conclusions drawn from the analysis.

**Table 5:** Fit indicators for LCAs modelling child abuse, neglect, and online child sexual solicitation and abuse

Model	Log-likelihood	resid. df	cAIC	BIC	aBIC	LMR-LRT *p*-value	BLRT *p*-value	Entropy	% small
1	−1853.300	57	3754.058	3748.058	3729.002	-	-	-	
**2**	**−1570.602**	**50**	**3244.030**	**3231.030**	**3189.741**	**< 0.001**	**0.006**	**0.803**	**14.57**
3	−1555.426	43	3269.046	3249.046	3185.525	< 0.001	0.434	0.76	5.89
4	−1542.090	36	3297.744	3270.744	3184.990	< 0.001	0.148	0.69	1.2
5	−1538.568	29	3346.067	3312.067	3204.081	0.424	0.834	0.615	1.3
6	−1535.108	22	3394.516	3353.516	3223.298	0.437	0.55	0.715	1.1

Note. Resid. df = residual degrees of freedom; cAIC = corrected Akaike Information Criterion; BIC = Bayesian Information Criterion; aBIC = sample-size adjusted Bayesian Information Criterion; LMR-LRT = Lo-Mendell-Rubin likelihood ratio test; BLRT = bootstrapped likelihood ratio test. A significant result, with a significance level of *p* <.050, indicates that the model with more classes fits the data better than the simpler model; % small = percentage of participants in the smallest group. Bold lines indicate the best-fitting model selected based on fit indices

**Fig. 1:**
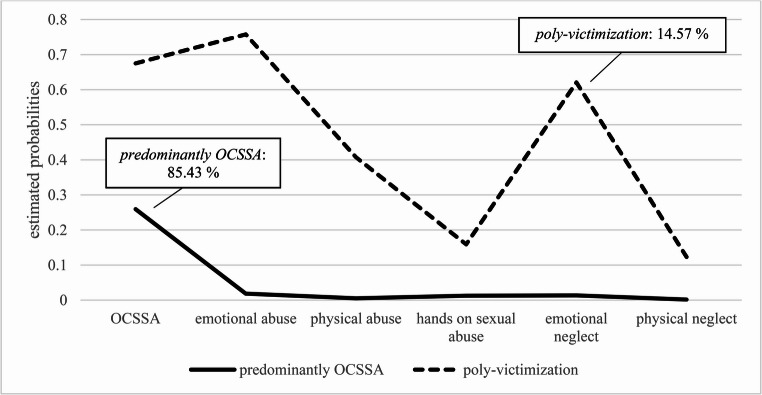
Profiles of the types of abuse for the two-class solution

Class 1 was labelled ‘*predominantly OCSSA*’, where the defining feature is the almost exclusive experience of OCSSA. In this class, other forms of offline child maltreatment have only very rarely been reported. Class 2 is described as ‘*poly-victimization*’ and includes individuals who have experienced multiple forms of online and offline maltreatment: OCSSA, emotional and physical abuse, sexual abuse as well as emotional and physical neglect. In addition to OCSSA, there are peaks in emotional abuse and emotional neglect within this class. Based on the highest posterior probabilities, 85.43% of the participants belong to class one and 14.57% to class two. Figure 1 shows the two identified classes, and Table 6 presents the descriptive characteristics of offline child maltreatment and OCSSA for both classes.

**Table 6:** Descriptive statistics of offline child maltreatment and OCSSA across the identified classes

	Class 1 ‘predominantly OCSSA’ *n* = 856	Class 2 ‘poly-victimization’ *n* = 146
	*n* (%)	*n* (%)
Any form of OCSSA	215 (25.1)	109 (74.7)
Any form of offline child maltreatment	43 (5.0)	146 (100.0)
Emotional abuse	17 (2.0)	114 (78.1)
Physical abuse	5 (0.6)	62 (42.5)
Hands-on sexual abuse	12 (1.4)	23 (15.8)
Emotional neglect	8 (0.9)	98 (67.1)
Physical neglect	1 (0.1)	19 (13.0)

### Descriptive statistics across the classes

There were no significant differences in gender or age by class membership (see Table 7). The analysis showed significant differences in class membership in terms of education, relationship status, and equivalence income. Individuals with a basic education were more frequently members of the poly-victimization class than those with an advanced education. Individuals who were in a relationship were more likely to be in the poly-victimization class than those who were not in a relationship. Individuals below the poverty risk threshold were more frequently members of the poly-victimization class than those above the poverty risk threshold. However, although significant differences in class membership based on education, relationship status, and equivalence income were observed, these differences had small effect sizes, suggesting that they may have limited practical significance.

**Table 7:** Socio-demographic differences between the two identified latent classes

	Class 1 ‘predominantly OCSSA’	Class 2 ‘poly-victimization’	Test-statistic
	*n* (%)	*n* (%)	
Class size	856 (85.4)	146 (14.6)	
Age (M, SD)	23.57 (3.536)	24.17 (3.318)	*U* = 56400.500, *Z* = −1.890, *p* =.059
Gender			
Female	417 (84.9)	74 (15.1)	*χ*^*2*^(1) = 0.172, *p* =.678, *V* = 0.013
Male	437 (85.9)	72 (14.1)	
**In a relationship**			
**Yes**	**323 (82.2)**	**70 (17.8)**	***χ***^***2***^**(1) = 0.6.575**, ***p*** **=.010**, ***V*** **= 0.082**
**No**	**522 (88.0)**	**71 (12.0)**	
**Highest level of education**			
**Basic education**	**532 (83.3)**	**107 (16.7)**	***χ***^***2***^**(1) = 6.614**, ***p*** **=.010**, ***V*** **= 0.081**
**Advanced education**	**323 (89.2)**	**39 (10.8)**	
**Equivalence income**			
**Below poverty risk threshold (<€1500)**	**164 (78.8)**	**44 (21.2)**	***χ***^***2***^**(1) = 8.426**, ***p*** **=.004**, ***V*** **= 0.093**
**Above poverty risk threshold (>€1500)**	**664 (86.9)**	**100 (13.1)**	

Note. OCSSA = online child sexual solicitation and abuse; U = Mann-Whitney U; Z = standardizes test statistic for the Mann-Whitney U test; *χ2* = Chi^2^; *V* = Cramer’s V; Bold indicates statistical significance at *p* <.050

## Discussion

To our knowledge, this is the first study to examine the co-occurrence of online child sexual solicitation and abuse with other forms of offline child maltreatment.

This study has shown that victims of OCSSA can be categorized into two latent classes: 85.39% of participants are classified as ‘predominantly OCSSA’ victims, while 14.61% experienced ‘poly-victimization’. The larger group of respondents with ‘predominantly OCSSA’ experiences (class 1) differs from the ‘poly-victimized’ group (class 2) in terms of the number of different types of abuse experienced. Class 1 thus represents a new group of affected individuals that has not yet been considered in the extensive research on victimization and the severe consequences of poly-victimization of ACEs [16, 36]. Respondents in class 1, who primarily experienced OCSSA, may have perceived these boundary violations (such as sexualized approaches or sexually suggestive questions) as part of adolescent curiosity and exploration of sexuality [37, 38]. Their natural curiosity may lead adolescents to use the internet as a platform to explore sexual content and relationships – a situation that can increase the risk of abusive encounters, particularly when they come across perpetrators who seek to exploit this curiosity [39, 40]. Current screening instruments used in clinical or therapeutic settings, such as the CATS-2 [41] or the UCLA Child/Adolescent PTSD Reaction Index for DSM-5 [42], do not adequately address OCSSA or include it in their assessments of traumatic experiences. As a result, there is a significant risk that individuals affected by online sexual abuse and solicitation will be missed. The high proportion of individuals affected by OCSSA, as demonstrated in our findings, underscores the importance of including questions about experiences of OCSSA, and thus media use, in regular diagnostic procedures and assessments. Class 2 is characterized by experiencing poly-victimization, with high rates of both online and offline maltreatment, specifically including OCSSA, emotional abuse, and emotional neglect. Experiencing multiple types of child maltreatment is the result of a complex interplay of various intra-individual, social and environmental factors. The pathways to poly-victimisation can be influenced by factors such as insecure community environments, family dynamics and personal emotional challenges. These factors can increase risk-taking behaviours and reduce an individual’s capacity for self-protection [43]. Our findings are consistent with previous studies that have consistently identified a group of poly-victimized individuals when examining risk profiles [18]. In addition to the well-researched forms of child maltreatment, our results show that this group is also affected by OCSSA. Demographic factors such as being female, non-heterosexual, and having parents with a lower level of education seem to be significant predictors of OCSSA. Furthermore, early offline sexual abuse was found to be the strongest predictor of OCSSA, both directly and indirectly through increased risky behaviour online [44]. These findings suggest that both online and offline maltreatment are associated with elevated risk behaviours, which in turn increase vulnerability to further forms of abuse. Therefore, recognising and addressing the interplay between online and offline victimization, and the associated risk factors, are crucial to the effective therapeutic and psychiatric treatment of individuals who have experienced multiple forms of maltreatment.

The results of this study highlight that attention must not only be directed toward poly-victimized individuals but also that parents need to be aware of those who have not yet been affected by offline abuse and neglect. The existence of a significant number of people who are exclusively victimised online suggests that different vulnerability mechanisms may exist. This may represent a distinct group of victims who have not previously been recognised, with different risk factors to those typically associated with offline abuse. Studies have shown that feelings of loneliness have increased, particularly among young people, as a result of the pandemic [45]. Spending more time online under these conditions may increase the risk of abuse and exploitation in digital spaces. Research shows that many parents do not fully understand what their children are doing online, with only a quarter of students affected by online bullying discussing these issues with their parents [46]. In the area of online sexual solicitation, studies have found that victims are more likely to report abuse to their peers rather than to their parents [47]. It is therefore possible that peer-prevention programmes could be an effective strategy in prevention efforts. Additionally, over half of U.S. parents report only sometimes knowing their children’s online activities [48]. This knowledge gap can prevent parents from recognizing the risks their children face, particularly those stemming from emotional abuse and neglect, which are often underestimated [49), 50]. Importantly, supportive adult relationships are a promotive factor for mental health [51].

The findings regarding class membership and its association with sociodemographic and -economic characteristics showed a relationship between poly-victimization and education, relationship status, and equivalence income. The overrepresentation of individuals with basic education is consistent with existing literature indicating a link between socioeconomic factors and vulnerability to multiple forms of maltreatment [43, 52]. As lower levels of education can contribute to less access to resources and support systems [53], it can exacerbate the impact of victimization experiences. The observed protective association of advanced education with predominantly OCSSA class membership may reflect the influence of social class, suggesting that these children may come from well-supported middle-class families with high levels of education who do not typically experience poly-victimization. Education can play a critical role in fostering resilience and awareness of potential risks in online and offline interactions. Higher educational attainment may also correlate with problem-solving skills and social support networks [54], which may serve as buffers against victimization. However, it is also possible that the relationship works in the opposite direction. Experiencing poly-victimization, particularly in childhood, may have long-term consequences that influence later socioeconomic status. Early adversity, such as abuse and neglect, may negatively affect educational attainment by disrupting cognitive development and school performance [55]. In addition, victimization-related stress and mental health problems can affect career opportunities and thus income levels in adulthood [56].

The results suggest an association between relationship status and class membership, with individuals being in a relationship being more likely to belong to the poly-victimization class. In this class, in addition to high rates of OCSSA, there was a prominent peak in emotional maltreatment. However, the data does not provide information about the quality or stability of these relationships. Being in a relationship does not necessarily mean that it is healthy or supportive. Previous experiences of emotional maltreatment can lead to problems forming secure and stable relationships. This aligns with research on attachment problems, which suggests that individuals with a history of emotional maltreatment are more likely to develop insecure attachment patterns [57,58]. Consequently, their relationships may be characterised by instability, conflict, or emotional dependency [59, 60]. Some people may seek out relationships as a way of coping with loneliness or a fear of abandonment, even if those relationships are dysfunctional. The desire to perpetually have a partner can indicate an underlying anxiety, frequently stemming from past experiences of neglect or abuse. Relationships may serve as a means of emotional regulation [61]. Previous research has also shown a link between childhood maltreatment and lower relationship satisfaction [62, 63]. Therefore, the higher likelihood of being in a relationship among individuals who have experienced multiple forms of victimisation should not be interpreted as a protective factor in itself, but rather as part of a complex pattern that may include elements of vulnerability.

Most studies on the prevalence of child maltreatment refer to samples of the adult population, as in many countries it is not possible to conduct surveys on sexual abuse among adolescents. These studies usually rely on retrospective self-reports, which can be affected by recall bias and differ in methodology regarding robustness and nature [65]. The present study, which surveyed the 18–29 age group in accordance with indicator 16.2.3 of the SDGs, provides child and adolescent psychiatry and psychotherapy with a highly relevant and timely insight into the actual burden of maltreatment experienced by young people today. Nearly one-fifth of young adults experienced at least one form of child maltreatment in this study. When examining the specific types of abuse experienced, the rates reported are about the same or lower than previously documented prevalences in another German study using the ACE questionnaire [15]: emotional abuse was slightly more common in the present study (2019: 12.5% vs. 2024: 13.1%), whereas both physical abuse (2019: 9.1% vs. 2024: 6.7%), sexual abuse (2019: 4.3% vs. 2024: 2.5%), emotional neglect (2019: 13.4% vs. 2024: 10.6%), and physical neglect (2019: 4.3% vs. 2024: 2.0%) were reported less in the present study. It should be noted, however, that this needs to be interpreted with caution as it compares a sample of 18–29-year-olds only with a population-based sample (14–96-year-olds), which may partly explain the differences. In addition, these comparisons are descriptive in nature and are not based on statistical significance tests. Further studies with samples exclusively comprising young adults are necessary to capture recent trends more accurately and to ensure more accurate conclusions [1, 19].

There has been a noticeable increase in the number of incidents of OCSSA, especially among the younger generation [1, 4], with prevalence rates reported to be almost twice that of offline sexual abuse [15, 66]. This stark difference highlights the shifting nature of abuse, where much of the exploitation takes place in private, digital spaces rather than physical environments. The increasing integration of digital technologies into everyday life has made the Internet indispensable, not only for education, but also for maintaining social relationships, navigating adolescence, and exploring sexuality [67, 68], making the Internet a key space for both, personal development and potential victimization. As the boundaries between online and offline experiences blur, the digital sphere has become a fertile ground for abuse, with perpetrators exploiting the anonymity and accessibility it offers [69].

### Implications for research and practice

This study represents a significant step forward in understanding the co-occurrence of OCSSA with other forms of child maltreatment. The stark differences in prevalence rates compared to previous studies highlight the urgent need for further research to explore the evolving dynamics of abuse in the digital age. As online environments have become key spaces for both personal development and potential victimization, researchers need to explore how these experiences intersect with traditional forms of abuse. Future research should differentiate between specific forms of OCSSA to better understand their unique dynamics, as there may be potential differences depending on, for example, whether the perpetrator is a peer or an adult. The consequences for mental well-being should be investigated, as should potential differences in the effects of online versus offline victimisation. Furthermore, future studies should explore resilience factors, as different victimisation profiles suggest that such factors may play a significant role throughout development [70]. It should also be explored what constitutes clearly abusive behaviour and what might be better understood as boundary-pushing behaviour. For example, unwanted sexualized questions, might not always be classified as abusive in offline contexts, depending on how the person initiating the advances respond to rejection [1, 71]. Such distinctions remain largely unexplored in online contexts.

Studying OCSSA requires complementary datasets. To gain a comprehensive understanding of the phenomenon, studies should encompass not only the 18- to 29-year-old age group, which sheds light on the entire childhood and adolescent period but also focus specifically on minors who are currently affected. Future research projects must address both age groups and explore how findings from these populations relate to and inform one another.

Practitioners should prioritize educating parents about the risks their children face in online spaces and encourage open communication about digital activities. Increasing parental awareness and understanding of online interactions can help bridge the knowledge gap and provide better support for children. In addition, understanding the different risk profiles of poly-victimized individuals can inform tailored interventions to address their specific needs. The findings also highlight the importance of fostering a social climate that encourages disclosure and reduces the stigma associated with discussing abuse, ultimately leading to more effective prevention and support strategies. When working with young people, mental health professionals should play an active role in identifying digital risks, including OCSSA. Asking about such experiences should become a routine part of the assessment process. In addition, promoting digital literacy is essential in order to help children and adolescents navigate the digital world safely and assess potential risks more effectively [72]. This study found that around one-third of participants had experienced at least one form of OCSSA. This highlights the urgent need to strengthen digital literacy efforts and raise awareness among mental health professionals. These professionals play a pivotal role in fostering digital awareness and resilience among young people. To fulfil this role effectively, they must possess the necessary digital skills to recognise potential online risks and provide informed, supportive guidance on how to manage them. Integrating OCSSA awareness into prevention, assessment, treatment, and intervention is essential to ensure that child protection, therapy, and counselling professionals can provide comprehensive care that reflects the realities of children’s online experiences.

### Limitations

The study has several limitations that should be considered when interpreting the results. First, the ACE measure of sexual abuse includes only contact acts, which may underestimate the prevalence of non-contact forms of abuse. This highlights the need for more comprehensive instruments that include digital and other non-contact forms of abuse, to capture the full spectrum of adverse childhood experiences in contemporary contexts. In addition, there are general criticisms of the ACE framework, particularly regarding its oversimplification of complex trauma experiences [73] as well as being primarily used in western countries [74]. Secondly, the study may not adequately represent marginalized groups, as the survey methodology may have excluded individuals who are less likely to participate in research due to various barriers, such as lack of language or institutional residency [75]. Thirdly, is the reliance on retrospective self-reporting, which is prone to recall bias and measurement error, particularly due to underreporting of adverse experiences [76]. A fourth limitation relates to the decision to exclude latent classes with a prevalence below 5%. This approach is methodologically justified to ensure replicability, since simulation studies have demonstrated that such small classes may represent unstable solutions [ 28]. However, balancing the need for stable, replicable classes with the possibility of overlooking rare but significant subgroups is a key consideration. Although excluding very small classes reduces the risk of spurious findings, it may also prevent important variations with practical or theoretical significance from being detected. Another methodological limitation of this study is the absence of external validation of class membership, which would have allowed for an assessment of concurrent validity. Although this was not feasible within the current study design, future research should aim to include such validation to strengthen the robustness of the latent class findings.

## Conclusion

In conclusion, this study provides a representative analysis of online child sexual solicitation and abuse and other forms of offline child maltreatment among young adults in Germany, revealing high prevalence rates that underscore the urgent need for awareness and intervention. The findings illustrate a significant shift in the nature of the abuse, with much exploitation occurring in private digital spaces, highlighting the challenges posed by the increasing integration of technology into everyday life. In addition, the distinct risk profile of victims, particularly those who primarily experience OCSSA, underscores the importance of understanding the psychological factors that contribute to their vulnerability online, as well as the consequences they may face.

To address these issues effectively, a focus on digital literacy is essential. Promoting education and communication, rather than control, in managing children’s and youth’s media behaviour can empower them to navigate the digital landscape more safely. It is critical to educate and train caregivers, parents as well as professionals about the risks of online sexual advances and abuse, and to ensure that they are equipped to help children identify and respond to potential threats. By fostering open communication and awareness, a safer online environment interaction can be modelled for young people to better protect them from exploitation. This requires individual-level approaches, targeted prevention efforts, and evidence-based interventions at the societal level, such as public education initiatives and school-based programmes. Given the substantial number of people affected by OCSSA, changes at the policy-making level are also necessary. This issue extends beyond isolated cases and affects a significant proportion of the population.

## Data Availability

Data and code are available from the corresponding author upon reasonable request.
